# Generation and gene expression profiling of 48 transcription-factor-inducible mouse embryonic stem cell lines

**DOI:** 10.1038/srep25667

**Published:** 2016-05-06

**Authors:** Kohei Yamamizu, Alexei A. Sharov, Yulan Piao, Misa Amano, Hong Yu, Akira Nishiyama, Dawood B. Dudekula, David Schlessinger, Minoru S. H. Ko

**Affiliations:** 1 Laboratory of Genetics, National Institute on Aging, National Institutes of Health, Baltimore, MD 21224, USA; 2 Department of Systems Medicine, Keio University School of Medicine, Tokyo 160-8582, Japan

## Abstract

Mouse embryonic stem cells (ESCs) can differentiate into a wide range – and possibly all cell types *in vitro*, and thus provide an ideal platform to study systematically the action of transcription factors (TFs) in cell differentiation. Previously, we have generated and analyzed 137 TF-inducible mouse ESC lines. As an extension of this “NIA Mouse ESC Bank,” we generated and characterized 48 additional mouse ESC lines, in which single TFs in each line could be induced in a doxycycline-controllable manner. Together, with the previous ESC lines, the bank now comprises 185 TF-manipulable ESC lines (>10% of all mouse TFs). Global gene expression (transcriptome) profiling revealed that the induction of individual TFs in mouse ESCs for 48 hours shifts their transcriptomes toward specific differentiation fates (e.g., neural lineages by *Myt1 Isl1*, and *St18*; mesodermal lineages by *Pitx1*, *Pitx2*, *Barhl2*, and *Lmx1*a; white blood cells by *Myb*, *Etv2*, and *Tbx6*, and ovary by *Pitx1*, *Pitx2*, and *Dmrtc2*). These data also provide and lists of inferred target genes of each TF and possible functions of these TFs. The results demonstrate the utility of mouse ESC lines and their transcriptome data for understanding the mechanism of cell differentiation and the function of TFs.

Pluripotent stem cells, such as embryonic stem cells (ESCs) are able to differentiate into many different cell types *in vitro*^1^. Because the ESCs are also immortal and can maintain their pluripotency, they can be inexhaustible research tools for investigating cell differentiation processes. Previously, we had established a “NIA Mouse ESC Bank” of 137 ESC lines, each of which carries a transcription factor (TF) that can be induced in a doxycycline-controllable manner^2^,^3^. We also carried out global gene expression profiling of these ESC lines 48 hours after the induction of TFs and demonstrated that these transcriptome data indicate the direction of cell differentiation^2^,^3^. In particular, we have validated the cell differentiation into neural lineages, skeletal muscles, hepatocytes, and blood cells^4^.

To increase the number of manipulated TFs and the coverage of cell types, we have generated ESC lines with 48 new transgenic TFs. With a total of 185 TFs, the “NIA Mouse ESC Bank” covers about 10% of all TFs encoded in the mouse genome^5^. We have measured the global gene expression profiles of these new ESC lines 48 hours after overexpressing each TF and compared the changes to tissue-specific gene expression profiles and functionally annotated gene sets.

## Results

### Generation of TF-inducible mouse ESC lines

**Figure 1:**
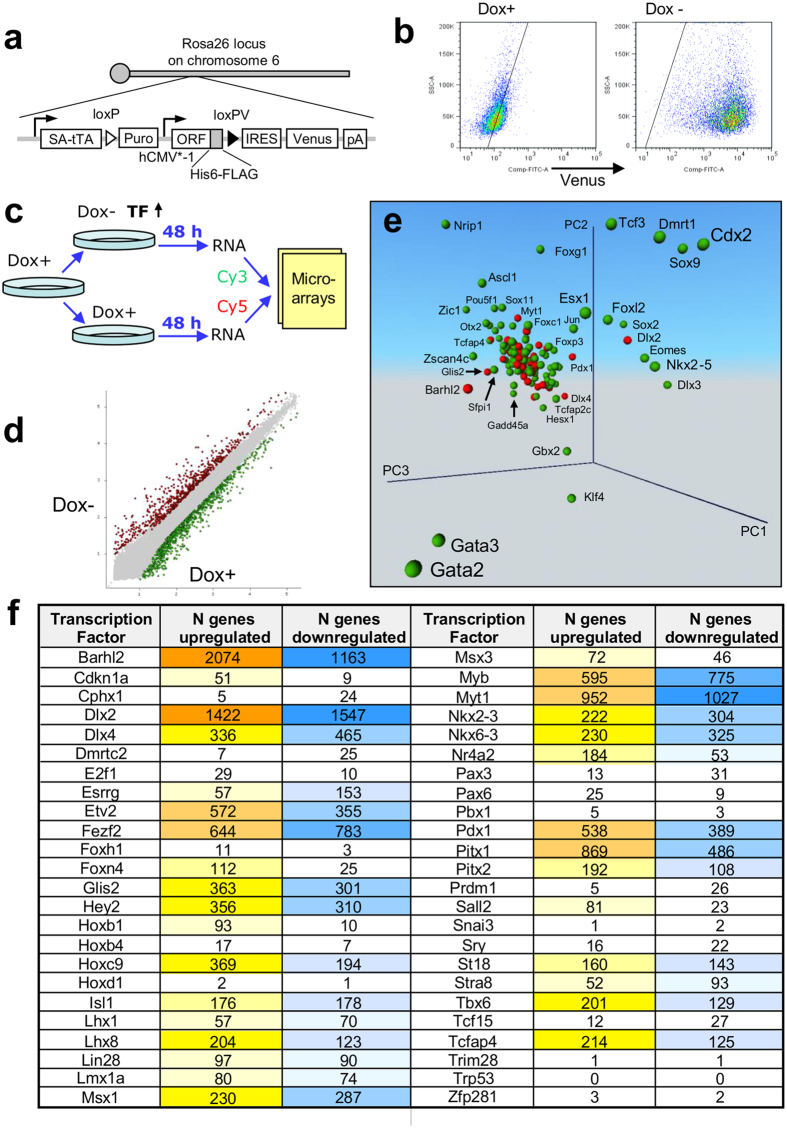
Induction of transcription factors (TFs) and its effect on the transcriptome. (**a**) Vector carrying a tetracycline-inducible (Tet-Off) transgenic TF was integrated into stably expressed Rosa26 locus in the genome. (**b**) Proportion of Venus-positive cells was evaluated by FACS (*Dlx4* clone). (**c**) Scheme of experiment: To activate the transgenic TF, Dox was removed from the media. Forty-eight hours later, RNA was collected from manipulated cells, and gene expression was quantified with microarrays via comparison with control cells that were continuously cultured in Dox+ conditions. (**d**) Example of a scatterplot comparing gene expression profiles with or without Dox for *Dlx2* induction. (**e**) Principal Component Analysis (PCA) of gene expression change in ES cells after induction of transcription factors; red – 48 new TFs analyzed in this paper; green – 137 TFs analyzed before^3^. Analysis is based on genes with significant change of expression (FDR ≤  0.05, change ≥ 1.5 fold). (**f**) Number of genes with significant change of their expression after the induction of individual transcription factors.

To generate TF-inducible ESC lines, we used the procedure reported previously^2^,^3^. The exogenous copy (transgene) of a TF integrated into the ubiquitously expressing Rosa26 locus^2^,^6^ is repressed by doxycycline (Dox), which is added to the culture medium., TF can then be activated by Dox removal (Fig. 1a). A Venus reporter included into the expression vector was used to visualize cells with the transgene induced (Fig. 1a,b). All tested clones showed at least 70% Venus-positive cells after the removal of Dox (Fig. 1b). The majority of the forty-eight new manipulated genes were TFs selected from a set of high-priority genes involved in important functions in mouse ES cells and their differentiation^7^.

To identify the effect of each TF on the transcriptome of ESCs, we used microarrays for gene expression profiling after 48 hours of culturing cells without Dox. Cells cultured in the presence of Dox were used as a control (Fig. 1c). The 48 hour time point was selected based on time-course experiments with multiple TFs^2^,^8^. This interval is sufficient to observe the change of expression in a large set of downstream genes, but short enough to observe a substantial enrichment of direct targets among responding genes. An example of a scatterplot with color-coded upregulated and downregulated genes after induction of *Dlx2* is shown in Fig. 1d. Principal Component Analysis indicated that the new set of tested TFs has, in general, weaker effects on the ESC transcriptome as compared to such TFs as *Gata2, Gata3, Cdx2, Nrip1, Dlx3, Ascl1, Gbx2*, and *Klf4* that were tested before (Fig. 1e).

The downstream effect of different TFs on gene expression was highly non-uniform, consistent with our previous studies^2^,^3^. Induction of 6 TFs (*Barhl2, Dlx2, Myt1, Fezf2, Myb,* and *Pitx1*) caused a substantial shift in the transcriptome: > 1,000 genes changed their expression (FDR ≤  0.05, fold change ≥ 1.5) (Fig. 1f). By contrast, 16 TFs had relatively minimal effects resulting in a change of expression in < 50 genes. For most TFs, the number of upregulated genes was comparable to the number of downregulated genes. However, some TFs acted mostly as activators (*Cdkn1a, Hoxb1, Foxn4, Foxh1, Sall2, Nr4a2, E2f1, Pax6*, and *Hoxb4*) and other TFs acted as repressors (*Prdm1, Cphx1, Dmrtc2, Esrrg, Pax3*, and *Tcf15*).

### Association of downstream genes of TFs with tissue-specific expression, gene ontology, and phenotypes

To explore the changes in the expression of downstream genes, we compared our microarray data with three databases: (1) GNF database ver. 3 on tissue-specific gene expression^9^,^10^; (2) Gene Ontology (GO) annotations^11^; and (3) Genetic Association Database (GAD) on gene sets associated with mouse phenotypes^12^. Because the GNF database is quantitative and the other two are qualitative, we used different methods to quantify association: (1) correlation of median-subtracted log-transformed gene expression values^3^, and (2) parametric analysis of gene set enrichment, PAGE^13^ (see Methods).

**Figure 2:**
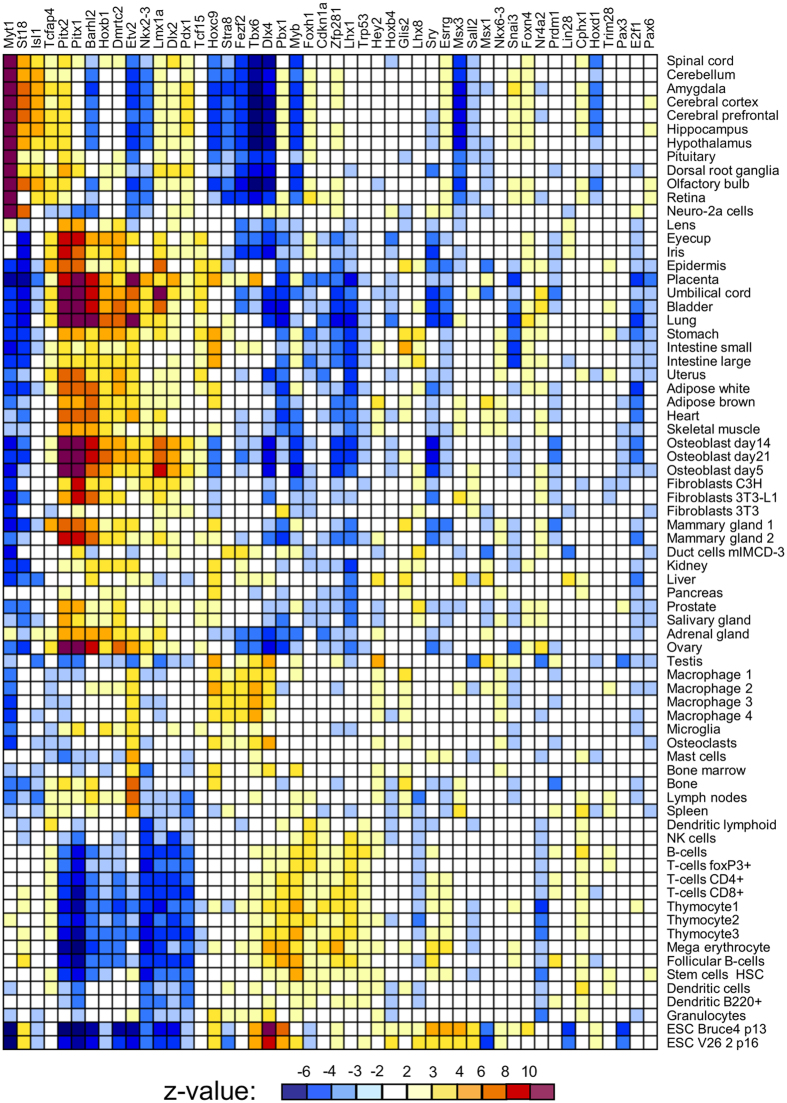
Correlation of gene expression response to the induction of transcription factors (this study) with tissue-specific gene expression from the GNF ver. 3 database; color shows z-value for correlation significance, white = non-significant correlation (z < 2).

**Figure 3:**
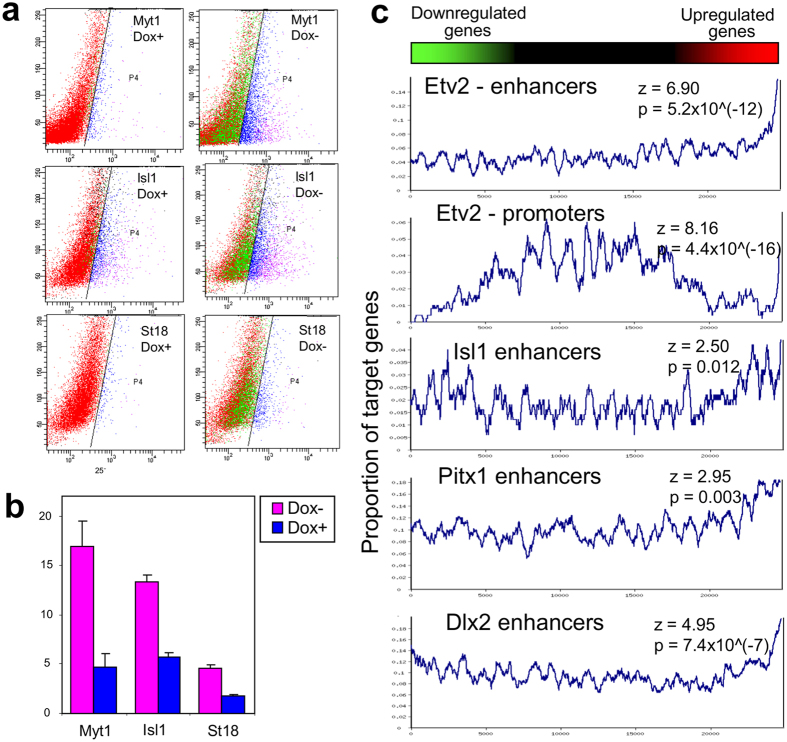
Validation of the capacity of TFs to facilitate ESC differentiation and activate target genes. (**a**) Analysis of the proportion of cells with neural-progenitor marker PSA-NCAM after induction of three TFs (Myt1, Isl1, and St18) for 6 days (3 days in α MEM abd 3 days in neuroCult) by FACS analysis; TFs were induced in Dox− conditions, whereas cells cultured in Dox+ conditions were used as control. (**b**) Average proportion of PSA-NCAM-positive cells after induction of three TFs in three replications. (**c**) Rank plot analysis for enrichment of target genes bound by transcription factors (TFs) in promoters (0–0.5 Kb from TSS) or enhancers (0.5–50 Kb from TSS) among genes upregulated after induction of these TFs. Genes were sorted by expression changes and then the proportion of target genes was estimated in a sliding window of 300 genes. Statistical significance was evaluated using PAGE^13^.

Comparison with the GNF database showed that the induction of individual TFs shifted the transcriptome toward specific differentiation fates. For example, gene expression change toward neural tissues was observed after induction of *Myt1, St18,* and *Isl1*; toward mesodermal lineages after induction of *Pitx1*, *Pitx2*, *Barhl2*, and *Lmx1a*; toward white blood cells after induction of *Etv2*, *My*b, and *Tbx6*; and toward ovary after induction of *Pitx1, Pitx2*, and *Dmrtc2* (Fig. 2). TFs associated positively with transcriptome changes toward specific lineages often showed a negative association with those toward different cell lineages. For example, effects of *Myt1* correlated positively with neural tissues but negatively with blood lineages (Fig. 2). Validation of the cell-differentiation potential of each TF is beyond the scope of this paper because it requires longer experiments (6–14 days) and is specific for each cell lineage^4^. As an example, however, here we provide information on the capacity of three TFs (*Myt1, Isl1*, and *St18*) to facilitate ESC differentiation towards neural fate. ESC clones with transgenic TFs were cultured in Dox− and Dox+ medium (3 days in α MEM and then 3 days in NeuroCult), and then the proportion of cells with neural progenitor marker PSA-NCAM was quantified by FACS (Canto II, Becton Dickinson). Induction of two TFs, Myt1 and Isl1, (in Dox− condition) resulted in a substantial increase in the proportion of PSA-NCAM(+ ) cells as compared to control (Dox+ condition) (Fig. 3a,b), which confirms that these TFs facilitate neural differentiation. The effect of St18 induction was too weak to score positively; it was somewhat higher than in controls (Dox+ ) for the same clone, but did not differ from controls in other two clones.

Analysis of GO gene sets showed that *Pitx1*, *Pitx2*, and *Barhl2* activated genes associated with collagen and skeleton; *Myt1*, *Hoxc9*, *Fezf2*, *Glis*2, and *Esrrg* activated synapse-related genes; *St18, Isl1, Dlx2, Dlx4. Lhx8,* and *Lmx1a* activated brain- and neuron-related genes; *Sall2* activated voltage gated ion channel-related genes; *Nkx2-3* and *Nkx6-3* activated eye-related genes; *Etv2* and *Pdx1* activated angiogenesis-related genes; *Tbx6* activated somitogenesis genes, *Sry* activated male sex determination genes, and *Lin28* and *Tcfap4* activated interferon-related genes (Table 1, Supplementary Table S1). Some of these associations (e.g., for *Etv2*, *Hoxc9*, *Nkx2-3*, *Pitx1*, *Sry*, and *Tbx6*) were the strongest among all 185 tested TFs. Additional information on the function of manipulated TFs was revealed via analysis of gene sets associated with mammalian phenotypes (GAD database). *Pitx1*, *Pitx2*, *Barhl2*, *Foxn4,* and *Hoxb1* activated skeleton-related genes; *Msx1* activated muscle and synapse-related genes; *Tbx6* activated chorion and heart-related genes; and *Dlx2*, *Lhx8,* and *Pdx1* activated ear-related genes (Table 1, Supplementary Table S2).

### Predicting direct targets regulated by TFs from gene expression change and TF binding

We tested whether genes upregulated after induction of TFs were enriched in the binding of TFs to promoters and/or enhancers, if such information on genome-wide binding (ChIP-seq) was available in the GEO database. Statistically significant enrichment (PAGE method) was detected for four TFs: *Etv2, Pitx1, Isl1*, and *Dlx2*, out of ten tested TFs (Fig. 3c). We used ChIP-seq data from mouse *Etv2*^14^, *Pitx1*^15^, *Isl1*^16^, and human *DLX2*^17^, because there was no ChIP-seq data on mouse *Dlx2*. The other six TFs tested (*Fezf2, Hoxc9, Msx1, Myb, Pitx2*, and *Prdm1*) did not show significant enrichment.

Target genes regulated by *Etv2, Pitx1, Isl1*, and *Dlx2* were identified using the method of Expected Proportion of False Positives (EPFP ≤  0.5, change ≥ 1.5 fold) (Supplementary Table S3)^18^. The largest set of regulated targets (*N* =  190) was found for *Etv2*; it was enriched in angiogenesis-related genes (GO:0001525), heart tube development-related genes (GO:0035050), and embryonic hemopoiesis-related genes (GO:0035162) (Supplementary Table S3). Regulated targets of *Dlx2* (*N *=  164) were enriched in genes associated with pituitary gland (GO:0021983), odontogenesis (GO:0042476), neurogenesis (for example, GO:0030182), and skeletal system (GO:0001501).

## Discussion

Systematic induction of individual TFs in undifferentiated ESCs followed by global gene expression profiling yields a useful resource for cell and molecular biology. It can identify TFs functioning upstream of any given gene, predict functional roles of TFs in cell differentiation, and select genes for potential application in gene therapy and regenerative medicine^2^,^3^. Correlation matrices of gene expression profiles between TF-induced ESCs and various tissues/organs can also provide candidate TFs, whose overexpression can induce the differentiation of ESCs into specific cell types, as we have shown in a proof of concept^4^. Further mining of the microarray results reported here as well as additional experiments with the ES cell lines and their derivatives could yield further insight into gene regulatory networks.

Previously published research provides a positive control for our bioinformatics-based functional analysis of gene expression change after induction of 48 transgenic TFs. For example, functions of *Myt1, St18,* and *Isl1* in neural tissues has been described^19^,^20^,^21^. TFs *Pitx1* and *Pitx2* are known to be involved in limb development^22^, consistent with our analysis of their downstream effects associated with mesoderm lineages. Roles of *Etv2* in angiogenesis is consistent between our analysis and published research^14^,^23^. Association of Myb with thymocytes has also been described^24^.

By contrast, the effects of some TFs were not anticipated. For example, *Barhl2* is known to function in the brain and spinal cord^25^,^26^, but in our data, the induction of *Barhl2* in mouse ES cells gave non-neural effects similar to *Pitx1* and *Pitx2*. As another example, *Tbx6*, which is known to determine neural and cardiac cell fate^27^, rather resulted in gene expression profiles trending toward macrophages (although GO annotations confirmed cardiac tendency as well). These discrepancies may point to additional unexplored functions of the TFs studied. Alternatively, or in addition, however, some effects observed in our experiments could be artifacts associated with the ectopic induction of TFs in the unusual context of ESC cultures in the medium employed. Thus, the unexpected results are both a caveat and a possible indication of new information.

Enrichment of TF binding in genes upregulated after the induction of *Etv2, Pitx1, Isl1*, and *Dlx2* is in accord with the expectation that downstream effects of TFs are likely to be mediated by TF binding to promoters and enhancers of their targets, which is the primary mechanism of their regulatory function. However, we cannot rule out additional effects of TFs, such as binding to other signaling molecules, protein modification, remodeling of chromatin, or indirect effects caused by an initial rapid activation of another TF(s) followed by a cascade of further gene activation.

In general, the preliminary analyses reported here provide indications that the collection of mouse ES cell lines reported here can be a starting point for more extensive attempts to form lineages and even tissues *in vitro*. As an example, we confirmed the capacity of Myt1 and Isl1 to enhance neural differentiation of ESCs. All transgenic ESC lines are freely available to the research community as a resource. Similar experiments for more regulatory genes (ideally for all TFs, signaling proteins, and non-coding RNA) should give increasingly complete information about selective gene regulation in mammalian systems. The approach can be further expanded via altering culture conditions, possibly including growth factors, or even the activation of multiple TFs simultaneously.

## Experimental Procedures

### Cell culture and microarray hybridization

ESC lines carrying a tetracycline-regulatable TF were derived from MC1 (129.3) cell line, which was obtained from the expanded frozen stock at Johns Hopkins University, as described previously^2^,^3^. ESCs of passage 25 were cultured in the standard LIF+ medium with added Dox+ on a gelatin-coated dish through the experiments. Cells from each cell line were split into six wells and the media was changed 24 hours after cell plating: three wells with Dox+ medium, and three wells with Dox− medium to induce transgenic TFs. Dox was removed via washing three times with PBS at three-hour intervals. The proportion of Venus-p;ositive cells was evaluated by FACS (Canto II, Becton Dickinson). Total RNA was isolated by TRIzol (Invitrogen) after 48 hours, and two replications were used for microarray hybridization. RNA samples were labeled with total RNA by Low RNA Input Fluorescent Linear Amplification Kit (Agilent). We hybridized Cy3-CTP labeled sample from Dox− medium together with Cy5-CTP labeled sample from Dox+ medium (i.e., control) to the NIA Mouse 44K Microarray v3.0 (Agilent, design ID 015087)^28^. Slides were scanned with Agilent DNA Microarray Scanner. All DNA Microarray data are available in Table S2, at GEO/NCBI (http://www.ncbi.nlm.nih.gov/geo; GSE72350), and at NIA Array Analysis, http://lgsun.grc.nia.nih.gov/ANOVA^29^.

### Neural differentiation of ESCs

For neural differentiation we used α MEM medium for 3 days followed by 3 days in the neuron-specific cell culture media: NeuroCult(TM) Differentiation Kit. NeuroCult(TM) NSC Basal Medium (Mouse), 450 mL (Catalog #05700), NeuroCult(TM) NSC Differentiation Supplements (Mouse), 50 mL (Catalog #05703). Differentiated cells were examined by flow cytometric analysis: after harvesting cells were stained with APC-conjugated PSA-NCAM antibody MoAb (Millteny Biotec) and then subjected to analysis by FACS Canto II (Becton Dickinson).

### Statistical analysis of gene expression data

Microarray data was log-transformed (log10) and normalized by feature intensity in control cells (Dox+ , no induction of TFs): *x*′ _*i*_ =  *x*_*i*_−*c*_*i*_ +  Median(*c*_*i*_), where *x*_*i*_ and *c*_*i*_ are log-transformed feature intensities in Dox− and Dox+ , respectively, in the array *i*. To combine new data with previous microarray results, we used batch normalization based on the median expression value of each gene. For statistical analysis, we used ExAtlas, which estimates the False Discovery Rate (FDR), to account for multiple hypothesis testing^30^. The response of genes to the induction of TFs was measured as a logratio (i.e., difference between means of logtransformed intensities) between manipulated (Dox− ) and control (Dox+ ) cells. We considered gene expression change as significant if logratio was significantly different from zero (FDR ≤  0.05) and the change of expression was ≥ 1.5 fold.

Correlation of gene expression changes induced by TF manipulation (i.e., logratio of Dox− vs. Dox+ ) versus tissue-specific gene expression in the GNF database (i.e., logratio of each tissue vs. median) was evaluated using ExAtlas^30^. The correlation analysis was done using 15,709 genes that were significant in both data sets. Criteria of significance for the GNF database were FDR ≤  0.05 and change ≥ 2 fold, which is higher than the 1.5 fold threshold used for our data on TF manipulation because the magnitude of gene expression difference between adult tissues was much greater than the magnitude of gene expression change after the induction of TFs. It was sorted first with hierarchical clustering, and then sorted manually.

Comparison of gene expression changes induced by TF manipulation with functionally annotated gene sets (i.e., GO, GAD, and sets of TF targets) was done using Parametric Analysis of Gene set Enrichment, PAGE^13^, implemented in ExAtlas^30^. PAGE was applied separately to upregulated genes (25% top genes sorted by logratio of Dox− vs. Dox+ ) and downregulated genes (25% bottom genes sorted by logratio). Sets of genes bound by TFs were identified from published ChIP-seq data for *Etv2* (GSM1436364, GSM1436365, GSM1436367, GSM1436367); *Pitx1* (GSM1019784, GSM1019786); *Isl1* (GSM782848, GSM928985, GSM928986); *Fezf2* (GSM1135048-GSM113504); *Hoxc9* (GSM766060, GSM766061); *Msx1* (GSM657516); *Myb* (GSM912903); *Pitx2* (GSM1162577); *Prdm1* (GSM1616574, GSM1616575); and *Dlx2* (GSM1208724). Peak coordinates were downloaded from the GEO database or from supplements to publications^31^,^32^. For some TFs, we filtered out peaks with low scores (< 100 for *Hoxc9*, <60 for *Pitx1*, <8 for *Pitx2*). If multiple samples were available, we used only matching peaks in at least three samples for Etv2 or two samples for other TFs. ChIP-seq peaks were annotated based on genomic coordinates of RefSeq and ENSEMBL transcripts downloaded from the UCSC database (http://genome.ucsc.edu). Transcripts were scored based on gene symbol (valid symbols were assigned a score of 3, whereas clones and predicted genes were assigned a score of 1) divided by distance from the peak to the transcription start site, TSS (distances < 1Kb were counted as 1Kb). Each peak was associated with one or two highest-score transcripts, and the second transcript was included if its score was >25% of the highest score. TF binding within 0.5 Kb from TSS was classified as a promoter, and binding within 0.5–50 Kb from TSS was classified as an enhancer.

## Additional Information

**How to cite this article**: Yamamizu, K. *et al.* Generation and gene expression profiling of 48 transcription-factor-inducible mouse embryonic stem cell lines. *Sci. Rep.* **6**, 25667; doi: 10.1038/srep25667 (2016).

## Supplementary Material

Supplementary Information

Supplementary Table S1

Supplementary Table S2

Supplementary Table S3

## Figures and Tables

**Table 1:** Summary table for the parametric analysis of gene set enrichment, PAGE^13^, for genes upregulated after induction of individual transcription factors*.

TF	Gene Ontology (GO)	Genetic Association Database (GAD)
Barhl2	Collagen, skeleton	Skeleton, aorta, limb
Dlx2	Neuron, ear, limb	Ear, limb, jaw
Dlx4	Gap junction, brain	Brain
E2f1	Chorion, neuropeptide	
Esrrg	Epithelium, synapse, estrogen	
Etv2	Angiogenesis, lymph vessel, heart	Embryo growth, aorta, neural crest
Fezf2	Neuron apoptosis, synapse	Brain, olfactory bulb, synapse
Foxn4	Brain, limb	Skeleton, vertebra
Glis2	Interferon, synapse	
Hey2	Symporter activity, interferon	Inflammation
Hoxb1	Skeleton, spinal cord	Neurogenesis, skeleton
Hoxb4	Pituitary	
Hoxc9	Synapse	Synapse, Purkinje cells
Isl1	Limb, sympathetic system	Limb, sympathetic ganglion
Lhx8	Ear, neuron	Ear, hippocampus, hypothalamus, hair
Lin28	Interferon	
Lmx1a	Brain, sympathetic system	Brain, cerebellum
Msx1	Retina, adrenal gland	Muscle, synapse
Msx3		Muscle, synapse
Myb	Germ cells	Myogenesis
Myt1	Heart, synapse, myelin	Nervous system
Nkx2-3	Eye, cytolysis	Eye
Nkx6-3	Eye	Lens, forebrain
Pax6	Calcium, face, renal system	Liver, ovary
Pdx1	Angiogenesis, brain	Ear, telencephalon
Pitx1	Collagen, skeleton, muscle, skin	Skeleton, teeth, ovary
Pitx2	Collagen, extracellular matrix, insulin	Skeleton, teeth, ovary
Sall2	Voltage gated ion channel	
Sry	Male sex determination	
St18	Hippocampus	
Tbx6	Somitogenesis, brain, heart	Chorion, heart
Tcfap4	Interferon, brain	Skin

^*^See Supplementary Tables S1 and S2 for details and statistics.
