# Supplementary material for: Generation and gene expression profiling of 48 transcription-factor-inducible mouse embryonic stem cell lines
Source: Sci Rep. 2016 May 6;6:25667. doi: 10.1038/srep25667 (PMC4858678; doi:10.1038/srep25667)
Supplement: Supplementary Information [file srep25667-s1.doc]

**Supplementary Data**

**Generation and gene expression profiling of 48 transcription-factor-inducible mouse embryonic stem cell lines**

Short title: Systematic induction of transcription factors in mouse ES cells

Kohei Yamamizu^1^, Alexei A. Sharov^1^, Yulan Piao^1^, Misa Amano^1^, Hong Yu^1^, Akira Nishiyama, Dawood B. Dudekula^1^, David Schlessinger^1^, and Minoru S.H. Ko^1,2,*^

^1^Laboratory of Genetics, National Institute on Aging, National Institutes of Health, Baltimore, MD 21224, USA

^2^Department of Systems Medicine, Keio University School of Medicine, Tokyo 160-8582, Japan

**Supplementary Table S1.** Parametric analysis of gene set enrichment, PAGE, for genes upregulated after induction of individual transcription factors and Gene Ontology (GO).

**Supplementary Table S2.** Parametric analysis of gene set enrichment, PAGE, for genes upregulated after induction of individual transcription factors and Genetic Association Database (GAD).

**Supplementary Table S3.** Targets of four transcription factors (TFs) upregulated after induction of these TFs. Information on the binding of TFs (ChIP-seq) was taken from GEO database (see Experimental Procedures). Regulated targets were identified using the following criteria: EPFP ≤ 0.5, and expression change ≥ 1.5 fold.
